# Epidemic Influences

**Published:** 1851-05

**Authors:** 


EPIDEMIC INFLUENCES.

We intended in this number to record the triumphs of sanitary science throughont the world, but have been unable to do so. The triumphs of sanitary science constitute one of the proudest trophies of medical science, and shows the philanthropy, benevolence, and science of the profession in a noble point of view. We hope to be able to make the sketch referred to in our July number. B.

